# Which Functional Classification Scale is Optimal for Children with Pulmonary Hypertension (PAH)?

**DOI:** 10.1007/s00246-020-02434-8

**Published:** 2020-08-09

**Authors:** Anna Migdał, Małgorzata Żuk, Dorota Jagiełłowicz-Kowalska, Zuzanna Powichrowska, Grażyna Brzezińska-Rajszys

**Affiliations:** Department of Cardiology, The Children’s Memorial Health Institute, Al. Dzieci Polskich 20, 04-730 Warsaw, Poland

**Keywords:** Pulmonary arterial hypertension, Children, WHO-FC, Pediatric functional classification

## Abstract

Functional status assessed by the WHO-FC scale derived from adults is a known prognostic factor for pulmonary hypertension. Data on the usefulness of the Panama-FC scale in assessing children with pulmonary hypertension are limited. The study was performed to compare functional status results (WHO-FC and Panama-FC) and to assess the usefulness of these scales in various clinical situations. The reliability of the Panama-FC questionnaire method for facilitating patient evaluation was also examined. 26 functional status assessments (7 in disease progression/after treatment intensification) in both scales were analyzed in 19 patients with PAH confirmed in RHC. WHO-FC, Panama-FC scales, and questionnaire-based on Panama-FC were conducted independently by three different physicians. Results of assessments were compared with each other and with 6MWD, NTproBNP level, and echo parameters (TAPSE, RV/LV ratio). The Panama-FC scale results obtained using the medical interview method and questionnaire did not differ. Both WHO-FC and Panama-FC classes well-reflected disease advancement confirmed by non-invasive parameters (NTproBNP, 6MWD, TAPSE, RV/LV ratio). Differences between grading the class in both scales were observed: 5pts were classified to II (Panama-FC) vs I (WHO-FC), 2pts were in lower risk group in WHO-FC (II) vs Panama (IIIa). Worsening or improvement after treatment intensification in functional status in both scales was connected with the significant change of NTproBNP level. The 6-min walking distance did not change. TAPSE, RV/LV ratio changed significantly in 3pts with IPAH, accordingly to change in WHO-FC and Panama-FC. WHO-FC and Panama-FC well reflect the disease advancement. The questionnaire method simplified the use of the Panama-FC scale. The Panama-FC scale appears to be better for assessing functional status during long-term follow-up, while the WHO-FC scale was more useful in short-term treatment monitoring.

**Electronic supplementary material:**

The online version of this article (10.1007/s00246-020-02434-8) contains supplementary material, which is available to authorized users.

## Introduction

Functional status is one of the basic biomarkers and prognostic factors in the management of PH patients [1]. Commonly used WHO-FC scale is based on four class NYHA classification and derived from an adult population. Evaluated symptoms include limitation of physical activity, dyspnea, fatigue, chest pain, and right heart failure. Such a scale is not always suitable for children due to not considering symptoms specific to pediatric patients. Therefore, adjusted to age modified Functional Classification for Children (Panama-FC) was created [2]. It was designed for five age groups (0–6 months, 6 months–1 year, 1–2 years, 2–5 years, and 5–16 years), and included five categories (I, II, IIIa, IIIb, IV). Each class consists of an evaluation of symptoms (similar to WHO-FC), but also appetite disorders, motor, and physical development and social functions. Therefore Panama scale is more complex and more difficult to use.

The aim of the study was to compare the results of functional status on both scales (WHO-FC and Panama-FC) and to evaluate the usefulness of these scales in various clinical situations.

The reliability of the Panama-FC questionnaire method for facilitating patient evaluation was also examined.

## Material and Methods

The clinical evaluation on both scales (WHO-FC and Panama-FC) was performed from April 2019 to January 2020 among 19 children under 16 years old with PAH. In all patients, PAH was confirmed by cardiac catheterization (mean pulmonary artery pressure ≥ 25 mmHg and PVRi > 3 Woods Um2). The study group consisted of patients aged 1.5–15.3 years (mean 10.1 ± 3.6, median 10.9) with different types of PAH: IPAH (*N* = 7), APAH-CHD (*N* = 9), multifactorial (*N* = 3).

Assessments were performed routinely every 3 months—at least 3 times during the study period. There were 14 clinically stable patients, in whom results on both scales and evaluated biomarkers remained unchanged. In those cases, only the first assessment was analyzed. Multiple assessments (2–3 per patient) were analyzed in five patients whose clinical condition had changed during the study period. Clinical worsening was defined as disease progression requiring treatment intensification (addition of a new medication or dose increase). Clinical assessment was performed in worsening and within 1–4 months after treatment intensification.

WHO-FC, Panama-FC, and Panama-FC questionnaire method assessments were conducted independently by three physicians. WHO-FC and Panama-FC evaluation were based on an medical interview with a patient and/or its parents, Panama survey evaluation was based on patient or parent's answers to the electronic questionnaire. The electronic survey based on the Panama-FC scale was designed to simplify the assessment method. Forms for five age groups (0–6 months, 6–12 months, 1–2 years, 2–5 years, 5–16 years) contained five adjusted to age questions about symptoms, appetite disorders, motor, and physical development and social functions (forms were added to supplementary materials). Responses were analyzed by the physician, then an appropriate class was selected.

The results of all first assessments (*N* = 19) in the WHO-FC, the traditional Panama-FC method, and the questionnaire were compared with each other and with 6MWD, NTproBNP level, echo parameters (TAPSE, RV/LV ratio). Additionally, 7 functional status assessments during clinical changes were analyzed.

Data were collected prospectively and contained results of examinations routinely performed in patients with pulmonary hypertension. For all procedures, informed consent from parents and older children was obtained.

## Results

Nineteen results of WHO-FC, Panama-FC, and Panama questionnaire obtained in the first assessment in all patients were analyzed.

Both WHO-FC and Panama-FC classes well-reflected disease advancement confirmed by non-invasive biomarkers (Tables 1, 2). The 6-min walk distance, NTproBNP level, and TAPSE changed accordingly with increasing functional class respectively. The RV/LV ratio increased significantly only in patients in a higher risk group (III or IIIa, IIIb).

**Table 1:** Dependence between functional status—WHO ( classes) and non-invasive biomarkers, mean value: NTproBNP (pg/ml); 6MWD (m); TAPSE (Z score); RV/LV (ratio)

WHO-FC	NTproBNP (pg/ml)	6MWD (m)	TAPSE (Z score)	RV/LV (ratio)
I	185	472	− 1.2	1.1
II	658	392	− 2.4	1.1
III	7263	386	− 3.8	1.4

**Table 2:** Dependence between functional status—Panama (classes) and non-invasive biomarkers, mean value: NTproBNP (pg/ml); 6MWD (m); TAPSE (Z score); RV/LV (ratio)

Panama-FC	NTproBNP (pg/ml)	6MWD (m)	TAPSE (Z score)	RV/LV (ratio)
I	177	516	− 1.0	1.1
II	476	422	− 1.3	1.1
IIIa	1728	376	− 4.1	1.4
IIIb	15,683	−	− 4.4	1.4

The Panama-FC scale results obtained using the medical interview method and the simplified questionnaire did not differ (Table 3)*.*

**Table 3:** Number of assessment in different classifications divided into classes

Class	WHO-FC	Panama-FC		Panama-FC questionnaire
I	9	4		4
II	7	10		10
III	3	IIIa	4	4
		IIIb	1	1

Two patients were classified into a higher risk group in the Panama-FC scale (IIIa) in contrast to the lower risk group in the WHO-FC scale (II). The difference between the classification resulted from consideration additional non-PH specific symptoms such as reluctance to eat, reluctance to efforts, delayed physical development, and individual teaching that could only be seen on the pediatric functional scale.

There was a difference between qualification for I and II class between the WFO-FC and Panama-FC scales. On the Panama-FC scale, five patients were rated in the lower class (II) in relation to delayed physical development (*N* = 1), reduced attendance at school (*N* = 2), in-depth interview on effort tolerance (*N* = 2).

Five patients were evaluated during disease progression and/or after treatment intensification to determine if both functional classes adequately reflected clinical changes. Characteristics of these patients, current treatment, changes in functional status, and non-invasive biomarkers are shown in Table 4. Clinical improvement after 1–4 months was connected with better results in both scales and associated with NTproBNP decrease. The worse functional class was observed during progression in both scales with a related increase in NTproBNP level. No significant changes in 6-min walk distance were found. Echocardiography parameters (TAPSE, RV/LV ratio) changed significantly in three patients with IPAH, accordingly to change in WHO-FC and Panama-FC.

**Table 4:** Changes in clinical status in five patients with relation to non-invasive biomarkers

	IPAH 1.5yo			IPAH 5.2yo			IPAH 10.9yo		APAH-CHD 12.1yo		APAH-CHD 13.1yo	
	0	TI	P	0	P	TI	0	P	0	TI	0	TI
Treatment	bos, sil	bos, sil, tre	bos, sil, tre	bos	bos	bos, sil	bos, sil, tre	bos, sil, tre	bos, sil	bos, sil, tre	bos, sil	bos, sil, il
Change from baseline (mth)		1	6.2		3	6.8		2.8		4		1.9
WHO-FC	III	**II**	IV	II	III	**I**	II	III	III	II	III	II
PANAMA- FC	IIIb	**IIIa**	IV	II	IIIb	**II**	II	IIIa	IIIa	II	IIIa	II
NTproBNP (pg/ml)	15,683	3415	22,800	558	4804	762	716	1216	5663	4748	443	378
6MWD (m)	–	–	–	370	420	420	420	470	315	300	456	445
TAPSE (z score)	− 4.4	− 2.5	− 5.5	− 1	− 3	0	0	− 1.5	− 2.4	− 2.6	− 4.6	− 3
RV/LV ratio	1.4	1.2	1.6	0.9	1.7	1.2	1.0	1.4	1.9	1.9	1.0	1.1

Mean value: NTproBNP (pg/ml); 6MWD (m); TAPSE (Z score); RV/LV (ratio), bolded- differences between scales

0—baseline, TI—after treatment intensification, P—progression, bos—bosentan, sil—sildenafil, tre—treprostinil, il—iloprost

Better results in the WHO-FC than in the Panama-FC scale were observed after treatment intensification in two patients. It was associated with impaired chronic development despite the absence of PH symptoms in one patient (I WHO-FC vs Panama-FC II), and due to the reluctance to eat associated with another disease with no severe symptoms of PH (WHO-FC II vs Panama-FC IIIa) in other.

## Discussion

Higher WHO-FC at diagnosis is well established prognostic factor in pediatric pulmonary hypertension [1]. Moreover, the WHO-FC deterioration as a symptom of the clinical worsening in the disease course has a strong predictive value for lung transplantation or death [3, 4]. Although, dedicated to pediatric population Panama-FC scale was proposed to use in 2011, data on the usefulness of the Panama-FC scale in assessing children with pulmonary hypertension are limited. Thus, WHO-FC is usually used in clinical practice and clinical trials as an endpoint despite its limitation in the pediatric population. Although this is a subjective assessment, WHO-FC is recommended for risk evaluation and as a treatment goal [4].

Our study demonstrates that the results of Panama-FC in children with pulmonary hypertension, similar to WHO-FC, well reflect disease advancement confirmed by other non-invasive biomarkers: 6MWD, TAPSE, and NTproBNP which are unquestioned prognostic factors in this disease [5, 6]. Due to a prospective character and short period of our study, the prognostic value of the Panama-FC scale could not be determined, a correlation between both scales and non-invasive biomarkers were analyzed only.

We notice a significant increase in RV/LV ratio in higher classes (III, and IIIa, IIIb) in both scales, which reflects the known mechanism of right ventricle adaptation to increased afterload in early stages of the disease, and right ventricle dilatation with altered systolic RV/LV interaction as hemodynamic changes progress [7].

To our knowledge, there is only one retrospective study assessing the usefulness of pediatric functional classification. Balkin et al. [8] concluded that Pediatric FC at diagnosis was not predictive of mortality, but a change in Pediatric FC during follow-up was associated with morbidity and mortality. They also evaluated compatibility between Pediatric FC and WHO-FC. Despite a strong agreement between both scales, higher Pediatric FC than WHO-FC was observed in 9/59 cases due to consideration of growth and developmental status. Similar results were observed in our study. The differences between the scales regarding worse Panama-FC results than WHO-FC were observed in more than 1/3 (7/26) assessments. This resulted from a more comprehensive evaluation of a patient in the Panama-FC scale, not only in terms of clinical symptoms but also in motor, physical, and social development.

This detailed assessment also affects follow-up results. The differences between scales during clinical improvement may result from slower changes in growth or development as well as from other concomitant diseases common in pediatric population, which are not considered in the WHO-FC. It was observed in 2/5 our patients whose clinical status had changed during the study. That’s why the WHO-FC scale seems to be better to evaluate rapid clinical improvement than Panama-FC.

Because the Panama-FC scale is more complex, it seems more difficult to use. We decided to simplify the original scale and change it into a questionnaire to facilitate the assessment and encourage its use. We observed strong agreement between evaluation in both methods: traditional and questionnaire. Moreover, by using the questionnaire method, any changes in a clinical evaluation are documented during follow-up, and features affecting the selection of the appropriate class can simply be observed. In such a subjective assessment, the impact of individual variables can be significant in determining the appropriate class.

In contrast to Balkin et al. [8], our study was conducted among patients with PAH only, with higher mean age, so we were limited by the small number of children in each age group (mostly 5–16 years). We did not take into account patients with pulmonary hypertension associated with lung diseases, in whom PH develops earlier and can be assessed on the Panama-FC scale dedicated to younger age groups (< 2 years old).

## Conclusions

Both scales (WHO-FC and Panama-FC) well reflect the disease advancement measured by objective tests.

The Panama-FC scale seems to be better for assessing functional status during long-term follow-up. However, other specific pediatric diseases may affect the results. Panama-FC questionnaire simplified the use of this scale.

The WHO-FC scale may be more useful to short-term monitoring after treatment intensification due to the lack of consideration of parameters changing over a longer period.

Based on our study, we recommend assessing pediatric PH patients on both scales, especially the Panama-FC scale for chronic treatment and the WHO-FC in short-term monitoring after treatment intensification.

## Study Limitation

Due to the small study group and short observational period our results are preliminary and require confirmation in a larger number of patients in longer follow-up. More data are needed to determine the impact of Panama-FC on prognosis.

## Electronic supplementary material

Below is the link to the electronic supplementary material.

Supplementary file1 (DOCX 16 kb) Questionnaire forms for each age group according to Functional Classification of pulmonary hypertension in children (Lammers et al)

## Abbreviations

6MWD: 6Minute walk distance
APAH-CHD: Pulmonary arterial hypertension associated with congenital heart disease
IPAH: Idiopathic pulmonary arterial hypertension
NTproBNP: N-terminal pro-brain natriuretic peptide
NYHA: New York Heart Association classification
PAH: Pulmonary arterial hypertension
PH: Pulmonary hypertension
PVRi: Pulmonary vascular resistance index
RHC: Right heart catheterization
RV/LV: Right-to-left ventricle diameter ratio
TAPSE: Tricuspid annular plane systolic excursion
WHO-FC: World Health Organization functional class
