# Supplementary material for: Which Functional Classification Scale is Optimal for Children with Pulmonary Hypertension (PAH)?
Source: Pediatr Cardiol. 2020 Aug 9;41(8):1725–9. doi: 10.1007/s00246-020-02434-8 (PMC7695665; doi:10.1007/s00246-020-02434-8)
Supplement: Supplementary file 1 — Supplementary file1 (DOCX 16 kb) Questionnaire forms for each age group according to Functional Classification of pulmonary hypertension in children (Lammers et al) [file 246_2020_2434_MOESM1_ESM.docx]

**SUPPLEMENTARY MATERIAL:**

**Questionnaire forms for each age group according to Functional Classification of pulmonary hypertension in children (Lammers et al)**

**Children 0-6 months**

1. Motor development (head control, rolls over, sitting with suport):

- Normal
- Delayed
- Inhibited, regression

2. Physical development (according to centiles)

- Normal
- Growing along own centiles
- Delayed

3. Nutrition

- Proper appetite and weight gain
- Impaired appetite
- Impaired appetite, supplementation or nutrition support

4. Physical activity

- No limitation of physical activity
- Slight limitation of physical activity
- Significantly reduced physical activity, reluctant to crawl. A quiet child, requires frequent naps

5. Symptoms

- No symptoms
- Shortness of breath, fatigued when playing
- Less than normal activity causes excessive fatigue, syncope
- Unable to carry out any physical activity without undue dyspnoea, fatigue or syncope Symptoms of right ventricular failure.

**Children 6-12 months**

1. Motor development  (crawling, sitting, grasping, starting to stand)

- Normal
- Delayed
- Inhibited, regression

2. Physical development (according to centiles)

- Normal
- Growing along own centiles
- Delayed

3. Nutrition

- Proper appetite and weight gain
- Impaired appetite
- Impaired appetite, supplementation or nutrition support

4. Physical activity

- No limitation of physical activity
- Slight limitation of physical activity, mobile child
- Significantly reduced physical activity, reluctant to play. A quiet child, requires frequent naps, feels comfortable at rest
- Total limitation of physical activity. A quiet child, requires frequent naps. Not interacting with family

5. Symptoms

- No symptoms
- Shortness of breath,  fatigued when playing
- Less than normal activity causes excessive fatigue, syncope
- Unable to carry out any physical activity without undue dyspnoea, fatigue or syncope Symptoms of right ventricular failure

**Children 1-2 years old**

1. Motor development (standing, starting to walk, climbing)

- Normal
- Delayed
- Inhibited, regressed

2. Physical development (according to centiles)

- Normal
- Growing along own centiles
- Delayed

3. Nutrition

- Proper appetite and weight gain
- Impaired appetite
- Impaired appetite, supplementation or nutrition support

4. Physical activity

- No limitation of physical activity
- Slight limitation of physical activity, mobile child
- Significant reduced physical activity: reluctant to play. A quiet child, requires frequent naps, feels comfortable at rest
- Total limitation of physical activity. A quiet child, requires frequent naps. Not interacting with family.

5. Symptoms

- No symptoms
- Shortness of breath,  fatigued when playing
- Less than normal activity causes excessive fatigue, syncope
- Unable to carry out any physical activity without undue dyspnoea, fatigue or syncope Symptoms of right ventricular failure.

**Children 2-5 years old**

1. Does the child attend to kindergarten?

- Regularly
- Attendance is about 75%
- Attendance is <50%
- Does not attend because of the severity of the disease
- Home schooling by choice

2. Physical development (according to centiles)

- Normal
- Growing along own centiles
- Delayed

3. Nutrition

- Proper appetite and weight gain
- Impaired appetite
- Impaired appetite, requires supplementation or nutrition support

4. Physical activity

- Unlimited, playing / attending sport with peers
- A slight limitation when exercising / attending sports with peers
- Significant reduced physical activity: not climbing stairs, reluctant to play with friends
- Significantly reduced physical activity: mobile at home, Wheelchair needed outside home.
- Total limitation of physical activity. Wheelchair dependant.  Not interacting with family, friends.

5. Symptoms

- No symptoms
- Dyspnoeic and fatigued when practicing sports/ playing with peers.  Feels comfortable at rest. No chest pain
- Less than usual activity (e.g. dressing) causes excessive fatigue, syncope, chest pain.  Feels comfortable at rest
- Unable to carry out any physical activity without undue dyspnoea, fatigue or syncope. Right ventricular failure

**Children 5-16 years old**

1. Does the child attend to school?

- Regularly
- Attendance is about 75%
- Attendance is <50%
- Does not attend because of the severity of the disease
- Home schooling by choice

2. Physical development (according to centiles)

- Normal
- Growing along own centiles
- Delayed

3. Nutrition

- Proper appetite and weight gain
- Impaired appetite
- Impaired appetite, requires supplementation or nutrition support

4. Physical activity

- Unlimited, plays sports, attends physical education with peers
- Slight limitation when exercising, playing sports with peers
- Significant reduced physical activity. Not playing sports, not attends physical education. Maintains  relationships  with  friends
- Significantly reduced physical activity: mobile at home. Wheelchair needed outside home
- Total limitation of physical activity. Wheelchair dependant.  Not interacting with family, friends.

5. Symptoms:

- No symptoms
- Dyspnoeic and fatigued when practicing sports/ playing with peers.  Feels comfortable at rest. No chest pain
- Less than usual activity (e.g. dressing) causes excessive fatigue, syncope, chest pain.  Feels comfortable at rest
- Unable to carry out any physical activity without undue dyspnoea, fatigue or syncope. Right ventricular failure
